# hMSH2 and GTBP expression in advanced stage epithelial ovarian cancer

**DOI:** 10.1038/sj.bjc.6690579

**Published:** 1999-07

**Authors:** A Ercoli, G Ferrandina, G Raspaglio, M Marone, N Maggiano, P Del Mastro, P Benedetti Panici, S Mancuso, G Scambia

**Affiliations:** Departments of Gynecology and Pathology, Catholic University of the Sacred Heart, L.go A Gemelli: 8-00168, Rome, Italy; Istituto di Ricerche di Biologia Molecolare, Pomezia, Italy

**Keywords:** hMSH2, GTBP, ovarian cancer, cisplatin

## Abstract

Defects in DNA mismatch repair have been associated with both hereditary and sporadic forms of human cancer. Most of the attention has been focused on the incidence and genetics of mismatch repair defects, while little is known about the expression levels of the mismatch repair proteins and their significance in cancer cell biology. In this study, both the expression levels of hMSH2 and GTBP proteins were investigated by Western blotting in 20 untreated epithelial ovarian cancers. For these analyses, a commercial anti-hMSH2 monoclonal antibody and a newly generated mouse monoclonal anti-GTBP antibody were used. hMSH2 and GTBP proteins were detected by Western blotting in 19 out of 20 (95%) samples analysed and were found to be directly correlated (*r* = +0.51, *P* = 0.025). hMSH2 expression was significantly higher in ovarian cancer cells originating from solid tumours than from ascites (*H* = 4.5, *P* = 0.033), whereas GTBP content did not significantly differ according to the origin of cancer cells. No statistically significant differences were found in the distribution of hMSH2 and GTBP levels according to the age of the patients, grade of differentiation, histotype and extent of surgical debulking. The amount of hMSH2 protein was demonstrated to be significantly lower in stage IV than in stage III patients (*H* = 7.35, *P* = 0.007). Moreover, significantly lower hMSH2 levels were observed in non-responding patients compared to patients who achieved complete or partial response to cisplatin-based chemotherapy (*H* = 4.88, *P* = 0.027). Conversely, GTBP levels were not distributed differently according to stage of disease and chemotherapy response. Our study suggests a possible involvement of hMSH2 in ovarian cancer cell biology and susceptibility to chemotherapy. The possible biological and/or clinical role of GTBP expression in ovarian cancer patients remains to be elucidated. © 1999 Cancer Research Campaign


*British Journal of Cancer* (1999) **80**(10), 1665–1671
© 1999 Cancer Research Campaign
Article no. bjoc.1999.0579

### hMSH2 and GTBP expression in advanced stage epithelial ovarian cancer

**A Ercoli[1], G Ferrandina[1], G Raspaglio[1], M Marone[1], N Maggiano[2], P Del Mastro[3], P Benedetti Panici[1], S Mancuso[1] and G Scambia[1]**

Departments of [1]Gynecology and [2]Pathology, Catholic University of the Sacred Heart, L.go A Gemelli: 8-00168, Rome, Italy; [3]Istituto di Ricerche di Biologia Molecolare, Pomezia, Italy

**Summary** Defects in DNA mismatch repair have been associated with both hereditary and sporadic forms of human cancer. Most of the attention has been focused on the incidence and genetics of mismatch repair defects, while little is known about the expression levels of the mismatch repair proteins and their significance in cancer cell biology. In this study, both the expression levels of hMSH2 and GTBP proteins were investigated by Western blotting in 20 untreated epithelial ovarian cancers. For these analyses, a commercial anti-hMSH2 monoclonal antibody and a newly generated mouse monoclonal anti-GTBP antibody were used. hMSH2 and GTBP proteins were detected by Western blotting in 19 out of 20 (95%) samples analysed and were found to be directly correlated ($r$ = +0.51, $P$ = 0.025). hMSH2 expression was significantly higher in ovarian cancer cells originating from solid tumours than from ascites ($H$ = 4.5, $P$ = 0.033), whereas GTBP content did not significantly differ according to the origin of cancer cells. No statistically significant differences were found in the distribution of hMSH2 and GTBP levels according to the age of the patients, grade of differentiation, histotype and extent of surgical debulking. The amount of hMSH2 protein was demonstrated to be significantly lower in stage IV than in stage III patients ($H$ = 7.35, $P$ = 0.007). Moreover, significantly lower hMSH2 levels were observed in non-responding patients compared to patients who achieved complete or partial response to cisplatin-based chemotherapy ($H$ = 4.88, $P$ = 0.027). Conversely, GTBP levels were not distributed differently according to stage of disease and chemotherapy response. Our study suggests a possible involvement of hMSH2 in ovarian cancer cell biology and susceptibility to chemotherapy. The possible biological and/or clinical role of GTBP expression in ovarian cancer patients remains to be elucidated.

**Key words:** hMSH2; GTBP; ovarian cancer; cisplatin

The DNA mismatch repair (MMR) system consists of a group of highly conserved genes that stabilize the cellular genome by correcting unpaired and mispaired bases during normal DNA replication and by blocking recombination events between divergent DNA sequences. Moreover, the human MMR system is involved in DNA repair of physically/chemically damaged DNA (Modrich and Lahue, 1996) and contributes to the control of the G2 cell cycle check point by recognizing certain types of DNA damage (Hawn et al, 1995). The relevance of the MMR system in stabilizing the genome is illustrated by the demonstration that MMR defective human cancer cell lines show a mutator phenotype characterized by increased DNA microsatellite instability and hypermutability of expressed genes (Boyer et al, 1995; Glaab and Tindall, 1997). Moreover, the disruption of this DNA repair pathway in germinal cells of individuals affected by hereditary non-polyposis colorectal cancer results in a strong predisposition toward tumour development. Finally, the phenotype associated with the loss of MMR function is similar to the phenotype resulting from the accumulation of somatic mutations in a great variety of sporadic human tumours (Eshleman and Markowitz, 1995). The MMR system in human cells is composed of at least five genes: *hMLH1*, *hMSH2*, *hMSH3*, *hPMS2* and *GTBP* (also known as *hMSH6*) that are strictly related to key components of the bacterial MutHLS mismatch repair system (for a review see Modrich and Lahue, 1996). The available evidence suggests that mismatch recognition in human cells is mediated by the two known mismatch recognition complexes: the hMSH2–GTBP heterodimer which acts on single base mispairs and loops of one or two bases and the hMSH2–hMSH3 heterodimer that binds preferentially loops of three and four bases (Acharya et al, 1996). The binding of these mispair recognition complexes to DNA is likely to form the substrate for interaction with other MMR protein complexes such as the hMLH1–hPMS2 heterodimer. The process is then completed by the excision and resynthesis of the DNA and ligation of the newly synthesized strand. Besides the well-established activity of the MMR system in correcting unpaired and mispaired bases, several in vitro studies have demonstrated that this DNA repair system can play a role in influencing tumour cell susceptibility to DNA-damaging cytotoxic agents (Branch et al, 1995; Hawn et al, 1995; Fink et al, 1996). It has been demonstrated that the hMSH2–GTBP complex and hMSH2 protein by itself are able to recognize and bind cisplatin (CP)-DNA intrastrand cross-links, which are the major DNA adducts produced following treatments of cells with CP (Duckett et al, 1996; Mello et al, 1996; Yamada et al, 1997). Moreover, Aebi et al (1996) reported that loss of the MMR genes results in acquired resistance to CP in two human ovarian cancer cell lines and that

*Received 18 May 1998*
*Revised 12 January 1999*
*Accepted 27 January 1999*

*Correspondence to:* G Scambia some human colorectal cell lines deficient in hMLH1 or hMSH2 are more resistant to CP than the sublines in which the MMR defect has been complemented by chromosome transfer. Finally, Brown et al (1997) suggested that loss of hMLH1 expression may be critically involved in the development of CP resistance in ovarian cancer patients. Although CP is widely used as one of the most effective chemotherapeutic agents for treating ovarian, testicular and several other solid tumours, the biochemical mechanisms underlying the responsiveness of cancer cells to this drug remain unknown.

To date, most of the attention has been focused on the incidence and genetics of MMR defects while little is known about the expression levels of the MMR proteins and their significance in human cancer cell biology.

The aim of this study was to investigate the expression levels of hMSH2 and GTBP proteins by Western blotting in a series of previously untreated ovarian cancer patients. The correlation between hMSH2 and GTBP levels and clinico-pathological characteristics and response to CP-based chemotherapy have been also investigated.

### PATIENTS AND METHODS

This study was conducted on 20 previously untreated primary ovarian cancer patients admitted to the Department of Gynecology at the Catholic University of Rome. Patient characteristics are listed in Table 1. The median age was 58 years (range 35–74). Fourteen patients had stage III and six had stage IV disease according to the International Federation of Gynecology and Obstetrics (FIGO) classification. One tumour was graded as well-differentiated (G1), three tumours as moderately differentiated (G2) and six as poorly differentiated (G3) (World Health Organization, 1979). Fifteen tumours were serous, three were endometrioid, one was mucinous and one was undifferentiated according to the World Health Organization histological typing of ovarian cancer (Serov and Scully, 1973). All patients underwent cytoreductive surgery. Surgical debulking was considered as optimal (residual tumour ≤ 2 cm) in 13 patients and non-optimal (residual tumour > 2 cm) in seven patients. Chemotherapy was instituted 2–3 weeks after surgery. All patients received chemotherapy containing CP (total CP dose = 500 mg). Gynaecological examination, abdominopelvic ultrasonography, CA-125 assay and radiological investigations, if necessary, were performed monthly for the clinical assessment of response, which was recorded according to the World Health Organization criteria (World Health Organization, 1979). Approximately 1 month after the last course of chemotherapy, clinically complete responders underwent second-look laparoscopy. In laparoscopy negative cases, second-look laparotomy was performed for the assessment of pathological response. Of 17 patients evaluable for chemotherapy response, nine showed a pathologically complete response, two showed partial response and six showed no change of disease or disease progression. In three clinically negative patients, pathological assessment of response was not carried out because of patient refusal of a second surgery.

### Generation of anti-GTBP monoclonal antibodies

The mouse anti-GTBP monoclonal antibodies (mAb) 66H6 and 21F10, both IgGγ1, were raised against the full-length recombinant GTBP protein. Female balb/c mice were immunized with four intraperitoneal and one intravenal injections. The spleen cells were then fused with the myeloma line P3χ63 Ag.8.653. Hybrids were selected with enzyme-linked immunosorbent assay (ELISA) and Western blots. They were then cloned by limiting dilution and the individual clones were again screened as above. The selected mAb-secreting lines were adapted to grow in roller bottles at a low percentage (1%) of fetal calf serum. The antibodies were purified from the culture medium by Gamma-plus Protein G Sepharose (Pierce).

**Table 1** Patient characteristics according to origin of the tumour cells and hMSH2 and GTBP levels

| Case | Origin of tumour cells | Age | Histotype | Stage | Grade | Residual tumour | Chemotherapy response | hMSH 2 levels (a.u.) | GTBP levels (a.u.) |
|---|---|---|---|---|---|---|---|---|---|
| 1 | Ascites | 49 | Serous | III | 3 | <2 cm | CR | 3.009 | 1.623 |
| 2 | Solid tumour | 68 | Serous | III | 3 | <2 cm | CR | 2.5 | 1.3 |
| 3 | Ascites | 60 | Serous | IV | 3 | >2 cm | CR | 1.244 | 1.376 |
| 4 | Solid tumour | 35 | Serous | III | 3 | <2 cm | CR | 4.044 | 2.546 |
| 5 | Solid tumour | 56 | Serous | III | 3 | >2 cm | CR | 2.255 | 0.506 |
| 6 | Solid tumour | 50 | Serous | III | 1 | <2 cm | CR | 2.357 | 0.224 |
| 7 | Ascites | 69 | Serous | III | 3 | <2 cm | CR | 2.15 | 1.88 |
| 8 | Ascites | 72 | Serous | III | 3 | <2 cm | CR | 1.406 | 1.765 |
| 9 | Ascites | 51 | Serous | IV | 3 | <2 cm | CR | 0.667 | 0.508 |
| 10 | Solid tumour | 44 | Serous | III | 3 | <2 cm | PR | 2.982 | 1.413 |
| 11 | Solid tumour | 60 | Endometrioid | III | 3 | <2 cm | PR | 2.175 | 0.611 |
| 12 | Ascites | 70 | Serous | III | 3 | <2 cm | NC-P | 0 | 0 |
| 13 | Solid tumour | 52 | Serous | IV | 3 | >2 cm | NC-P | 0.389 | 0.28 |
| 14 | Ascites | 74 | Undifferentiated | IV | 3 | >2 cm | NC-P | 0.852 | 0.961 |
| 15 | Solid tumour | 46 | Serous | IV | 3 | >2 cm | NC-P | 1.543 | 0.427 |
| 16 | Ascites | 42 | Serous | III | 2 | <2 cm | NC-P | 1.092 | 1.624 |
| 17 | Ascites | 63 | Serous | III | 3 | >2 cm | NC-P | 1.557 | 1.6 |
| 18 | Ascites | 69 | Endometrioid | IV | 3 | >2 cm | n.d. | 1.63 | 1.112 |
| 19 | Ascites | 54 | Endometrioid | III | 2 | >2 cm | n.d. | 0.916 | 0.576 |
| 20 | Solid tumour | 65 | Mucinous | III | 3 | >2 cm | n.d. | 2.28 | 1.174 |

CR: complete response; PR: partial response; NC-P: no change of disease or disease progression; N.D.: not determined.

 *© 1999 Cancer Research Campaign*

### Preparation of primary tumour cells

Tumour specimens and ascitic fluid were aseptically obtained during surgery and tumour cells were prepared as previously described (Scambia et al, 1992) with minor modifications. Briefly, tumour cells were separated from ascitic fluid by centrifugation and from solid tumour biopsies by mechanical and biochemical (0.1% collagenase IV, Sigma, Milano, Italy) dissociation in Ham's F-12 medium supplemented with antibiotics under aseptic conditions in a laminar flow hood. Cells were then filtered through a sterile gauze to remove cell clumps and passed trough 25-gauge needles in order to obtain a monocellular suspension. Cells were separated on a Ficoll-hypaque gradient. Collected cells were extensively washed in Hank's balanced salt solution and resuspended in Ham's F-12 medium. Tumour cells, assessed by immunohistochemical staining for cytokeratin on cytospin preparations, were detected in a percentage ranging from 64 to 92% (median: 73%).

### Cell lines

The MMR-proficient human ovarian carcinoma cell line A2780, the human cervical cancer cell line HeLa, and the MMR-deficient human colorectal carcinoma cell lines LoVo (*hMSH2*-deficient) and HCT-15 (*GTBP*-deficient) (Boyer et al, 1995) were used in this study. All cultures were kindly provided by Dr P Karran (Imperial Cancer Research Fund, Herts, UK) and were maintained in RPMI-1640 medium supplemented with 10% fetal calf serum, 100 U $ml^{-1}$ antibiotics and 0.3 μg $ml^{-1}$ glutamine in a humidified 5% carbon dioxide incubator.

### Preparation of cell lysates and Western blotting analysis

Total cellular proteins were isolated blind from primary tumour cells and from cell lines harvested during exponential growth, by lysing the cells in 1% sodium dodecyl sulphate (SDS), 20 mM EDTA, 10 mM Tris–HCl pH 8, 1.74 μg $ml^{-1}$ phenylmethylsulphonyl fluoride, 2.5 μg $ml^{-1}$ leupeptin, chymostatin, pepstatin and 0.2 μg $ml^{-1}$ aprotinin. For hMSH2 and GTBP detection, 250 μg of each protein sample in 1 × SDS sample buffer were separated onto a 6% SDS-polyacrylamide gel. After electroblotting, the polyvinylidene difluoride (Millipore Co., Bedford, MA) membranes were incubated with 6% non-fat dry milk in 1 × TBST (0.1 M Trizma base, 0.15 M sudium chloride, 0.05% Tween-20, pH 7.4) for blocking and then with the primary antibody in 3% non-fat dry milk in 1 × TBST. Following incubation with an alkaline phosphatase-conjugated goat anti-mouse antibody (Bio-Rad Laboratories, Hercules, CA, USA), visualization of the bound antibody was performed with the BCIP/NBT Phosphatase Substrate System (Kirkegaard & Perry Laboratories, Gaithersburg, MD, USA). The samples were also analysed by Western blot for α-tubulin. Images of the blots were acquired with a Cohu CCD camera and quantification of the bands was performed by Phoretix 1 D (Phoretix International Ltd, Newcastle upon Tyne, UK). Band intensity was expressed as relative absorbance and hMSH2 and GTBP values were normalized to α-tubulin levels. Two to four different Western blot analyses were performed on our samples. The variability observed was never greater than 20%. The following mAbs were used: amino-terminal Ab-1 mouse anti-human MSH2 (Oncogene Science, Cambridge, MA, USA) (at 1:100 dilution), Ab-1 mouse anti-human α-tubulin (Oncogene Science, Cambridge, MA, USA) (at 1:100 dilution) and amino-terminal 21F10 and 10E10 mouse anti-human GTBP (generated as described above) (at 1:500 dilution).

### Immunohistochemical analysis of hMSH2 in tissues and cell lines

Immunohistochemical analysis was performed using Ab-1 anti-hMSH2 antibody on frozen sections from histologically defined neoplastic ovarian tissues, normal human colonic mucosa and on cytospins of logarithmically growing A2780, LoVo and HCT-15 human cancer cell lines. Frozen tissues were obtained from surgical resections and stored at –80°C until used. Cryostat sections and cytospins were placed on SuperFrost slides and fixed in 4% paraformaldehyde for 10 min at room temperature. Endogenous peroxidase was blocked with 0.5% hydrogen peroxide in absolute methanol for 30 min. The primary antibody was applied for 1 h in 1:100 dilution and sequentially followed by biotinylated anti-mouse immunoglobulin and horseradish peroxidase-conjugated streptavidin (ABC-Vector Laboratories, Burlingame, CA, USA) and then incubated in the chromogenic substrate solution 3,3′-diaminobenzidine (Sigma, Milano, Italy) for 10 min. Sections were lightly counterstained with Harris's haematoxylin, dehydrated, cleared and mounted. The percentage of positivity of the neoplastic cells was evaluated by counting at high magnification (1000 ×) at least five different areas of the samples. Sample analysis was performed blind.

### Statistical analysis

Pearson's correlation test was used to analyse the relationship between hMSH2 and GTBP levels. Kruskal–Wallis non-parametric test was used to analyse the distribution of hMSH2 and GTBP protein levels according to the clinico-pathological characteristics of the patients.

### RESULTS

### Characterization of anti-GTBP antibodies in cell lines

Among the anti-GTBP mAbs tested, 21F10 exhibited the best specificity and reactivity. Western blot analysis performed with 21F10 showed a main band of approximately 160 kDa corresponding to full-length GTBP in lysates from MMR-proficient A2780 and HeLa cell lines and in the *hMSH2*-defective LoVo cells (Figure 1). The amount of GTBP was considerably lower in LoVo lysate than in A2780 and HeLa lysates. Full-length GTBP was not detectable by Western blotting in the HCT-15 cells (Figure 1) which contain frameshift mutations in *GTBP* gene resulting in truncated proteins (Papadopoulos et al, 1995). However, this cell line showed a marked immunohistochemical nuclear reactivity with 21F10 anti-GTBP mAb (data not shown). This finding could indicate the preserved immunoreactivity of the truncated GTBP proteins since the frameshift mutations which generate a termination codon in HCT-15 cells (Papadopoulos et al, 1995) did not affect the amino-terminal region recognized by our mAb. In the absence of a characterized negative control for GTBP immunostaining in the literature, we considered the 21F10 anti-GTBP mAb as inadequate for precise immunohistochemical assessment of this protein.

© 1999 Cancer Research Campaign

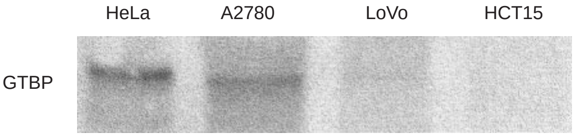

**Figure 1** Western blot analyses of A2780, HeLa, LoVo and HCT-15 cell lines performed with 21F10 mouse anti-human GTBP mAb. A main band of about 160 kDa, corresponding to full-length GTBP, is detected in the MMR proficient A2780 and HeLa and in the *hMSH2*-defective LoVo cell lines. The amount of GTBP was considerably lower in LoVo than that in A2780 and HeLa cells. No detectable levels of GTBP were found in the *GTBP*-defective HCT-15 cells

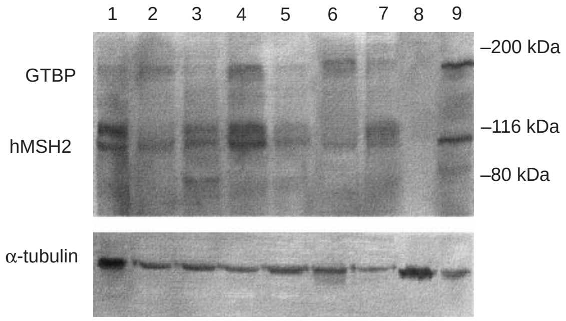

**Figure 2** Representative Western blot analysis of hMSH2 and GTBP with Ab-1 and 21F10 mAbs of lysates of purified ovarian tumour cells (lanes 1–8) and of A2780 ovarian cancer cell line (lane 9). A band of 105 kDa, corresponding to hMSH2, and a band of 160 kDa, corresponding to GTBP, are observable in all samples but one (lane 8). In the lower panel, the samples were probed for α-tubulin

### Western blot analysis of hMSH2 and GTBP in ovarian cancer cells and relationship with clinico-pathological parameters

The expression levels of hMSH2 and GTBP in ovarian tumour cells obtained from ascites ($n$ = 10) and solid tumours ($n$ = 10) were examined by Western blot analysis with Ab-1 anti-hMSH2 and 21F10 anti-GTBP mAbs. A band of approximately 105 kDa, corresponding to hMSH2 and a band of 160 kDa, corresponding to GTBP (Figure 2) were observed in 19 out of 20 (95%) samples analysed. A single case (ascitic cancer cells) did not show any detectable band even when a twofold greater amount of protein lysate was analysed (Figure 2, lane 8). This case was not considered in all statistical analyses described below. An additional fainter band of approximately 80 kDa was observed in some samples analysed with the anti-hMSH2 mAb (Figure 2, lanes 3 and 9). This band has been previously described by Mello et al (1996) and is believed to be a specific degradation product of the hMSH2 protein. Moreover, a slower migrating band of approximately 120 kDa was observed in Figure 2, lanes 1, 3, 4, 5 and 7: at present, we are unable to explain the nature of this band and no information about possible post-translational modifications of hMSH2 is currently available. Table 1 shows hMSH2 and GTBP levels according to the origin of tumour cells and the clinico-pathological characteristics of the cases examined. The densitometric values of the hMSH2 and GTBP bands were normally distributed and ranged from 0.389 to 4.044 (median 1.63) absorbance units (a.u.) for hMSH2 and from 0.224 to 2.546 (median 1.174) a.u. for GTBP. hMSH2 and GTBP levels were found to be directly correlated ($r$ = +0.51, $P$ = 0.025) (Figure 3). hMSH2 expression was significantly higher in ovarian cancer

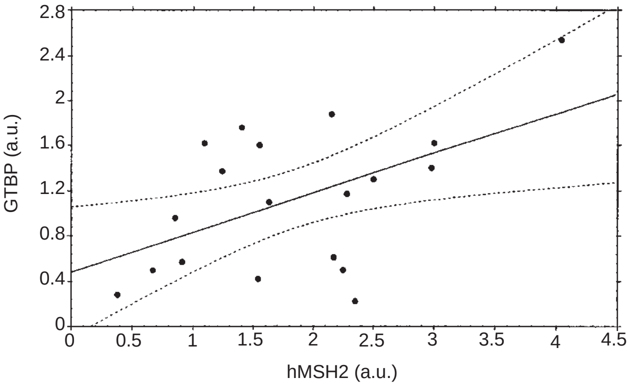

**Figure 3** Correlation between hMSH2 and GTBP levels in primary ovarian cancer cells cells originating from solid tumours than from ascites ($H$ = 4.5, $P$ = 0.033), whereas GTBP content did not significantly differ in solid versus ascitic cancer cells ($H$ = 0.96, $P$ = 0.32).

The clinico-pathological characteristics and chemotherapy response of the patients were not distributed differently according to the origin of the tumour cells. No statistically significant differences were discovered in the distribution of hMSH2 and GTBP levels with respect to the age of the patients, grade of differentiation (G1–2 vs G3), histotype (serous vs non-serous) and extent of surgical debulking (optimal vs non-optimal) (data not shown). hMSH2 protein levels were significantly lower in stage IV patients than in stage III patients ($H$ = 7.35, $P$ = 0.007) while GTBP tended to be expressed at lower levels in stage IV cases ($H$ = 3.01, $P$ = 0.08). Significantly lower hMSH2 levels were found in cancer cells from non-responding patients than in cancer cells from patients who achieved complete or partial response to CP-based chemotherapy ($H$ = 4.88, $P$ = 0.027). GTBP levels were not differently distributed according to chemotherapy response (data not shown).

### Immunohistochemical analysis of hMSH2 in cancer cell lines and neoplastic ovarian tissues

Immunohistochemical analysis of hMSH2 with Ab-1 mAb was performed in the MMR-proficient A2780 ovarian cancer cells, in the *hMSH2*-defective LoVo colon carcinoma cells and in six ovarian cancer specimens obtained from primary surgery from a corresponding number of untreated ovarian cancer patients. Over 90% of A2780 cells exhibited an intense staining localized exclusively in the nuclei while LoVo cells, which carry a deletion in both alleles of the hMSH2 gene (Umar et al, 1994), did not show immunoreactivity (Figure 4 A and B respectively). In all samples of primary ovarian cancer, a specific nuclear immunoreaction with Ab-1 was observed in the vast majority of the neoplastic cells (over 80% of positive cells) (Figure 4 C, D). In three specimens of normal human colonic mucosa used as a positive control for Ab-1 staining, immunohistochemistry revealed that hMSH2 immunoreactivity was confined to the glandular epithelium (data not shown) as previously described by several authors using different anti-hMSH2 antibodies (Wilson et al, 1995; Leach et al, 1996).

### DISCUSSION

This is the first study analysing the expression levels of hMSH2 and GTBP proteins in a series of primary ovarian cancers. We

© 1999 Cancer Research Campaign

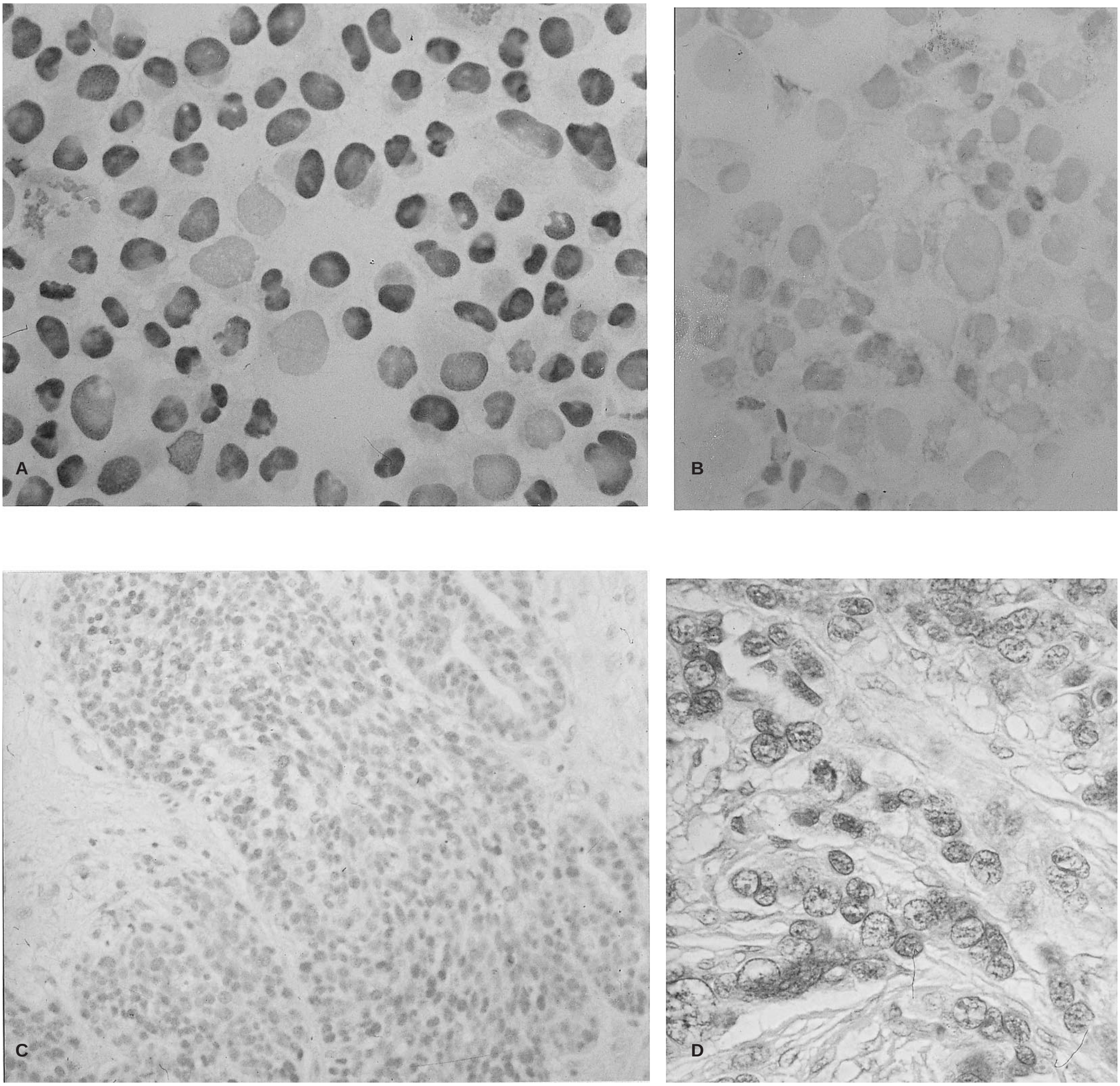

**Figure 4** Immunocytodetection (at magnification of 1000 ×) of hMSH2 protein with Ab-1 mAb in the MMR-proficient A2780 ovarian cancer cells (**A**) and in the *hMSH2*-defective LoVo colon carcinoma cells (**B**). Over 90% of A2780 cells exhibited an intense staining exclusively localized in the nuclei while LoVo cells did not show immunoreactivity. Representative immunohistochemical analysis of hMSH2 protein with Ab-1 mAb in primary ovarian cancer (**C** at magnification 200 ×, **D** at magnification 1000 ×). A specific nuclear immunoreaction for hMSH2 is present in over 80% of neoplastic cells demonstrated that hMSH2 protein is exclusively localized in the nuclear compartment and is expressed in the vast majority of neoplastic cells from primary ovarian tumours. These results are consistent with those of Brown et al (1997), and with previous demonstrations that microsatellite instability occurs with a low prevalence in advanced stage ovarian cancer (King et al, 1995; Arzimanoglou et al, 1996), indicating that the majority of tumour cells from these patients carry a functional *hMSH2* genes. Western blotting analysis revealed that hMSH2 and GTBP display a wide range of expression levels, suggesting that both these proteins could play a role in the biology of ovarian tumour cells.

It has been previously demonstrated by Palombo et al (1995) that the amount of hMSH2 and GTBP in the *GTBP*-deficient HCT-15 and in the *hMSH2*-deficient LoVo cells, respectively, was considerably lower that that in the MMR-proficient HeLa cells, thus leading these authors to hypothesize that the two proteins are unstable when not in a complex. Even though we cannot rule out this possibility, the direct correlation between hMSH2 and GTBP expression levels found in primary ovarian cancer cells is likely to suggest that the expression of these two proteins may be regulated by a common mechanism.

Only hMSH2 levels were found to significantly differ according

*© 1999 Cancer Research Campaign*

to the origin of the tumour cells, suggesting that hMSH2 levels could be related to different biological characteristics of ascites-derived with respect to solid tumour-derived cells. Ovarian tumour cells which exfoliate from the surface of the solid tumour are likely to develop distinct biological characteristics since they lose cell–cell and cell–substrate interactions and, on the other hand, develop the ability to grow in the intraperitoneal microenvironment, which is particularly rich in cytokines and growth factors (Kutteh and Kutteh, 1992), and to form metastatic implants on peritoneal surface. However, in order to investigate whether primary tumour and ascites represent two biologically distinct entities, a multivariate analysis including other biologically relevant parameters is needed and thus a larger series of cases is required to adequately address this issue.

No statistically significant differences were found in the distribution of hMSH2 and GTBP levels according to age of the patients, grade of differentiation, histotype and surgical debulking. Conversely, we found that low hMSH2 protein levels were associated with poor chance of response to CP-based regimens. Our findings cannot be seen merely as a consequence of the reduced hMSH2 levels in stage IV patients since no difference in the type of response according to stage of disease was found in our series. Two different hypotheses could be considered: first, it has been demonstrated that increased hMSH2 protein expression is associated with the entrance of resting cells into the cell cycle (Marra et al, 1996). It is therefore conceivable that cancers expressing low hMSH2 levels have a low proliferative fraction, which is commonly considered to imply a low susceptibility to chemotherapy. Alternatively, it has been demonstrated that the amount of hMSH2 protein directly correlated with mismatch G-T binding activity and that both parameters inversely correlated with the level of drug resistance in various Chinese hamster ovary cells showing different degrees of resistance to methylating agents (Dosch et al, 1998). In this context, Mello et al (1996) previously hypothesized that, similarly to the biochemical pathway proposed for methylating agents, a mechanism that involves a futile cycle of excision and resynthesis of damaged DNA and ultimately results in cell death, could also be operative in the processing of CP adducts. According to this model, the binding of hMSH2 to CP-modified DNA would trigger the recruitment of other MMR protein(s) causing misdirected repair attempts at sites of CP damage. This abortive repair activity and/or the subsequent accumulation of DNA strand breaks could provide a signal resulting in growth arrest in the G2 phase of the cell cycle and subsequently cell death. Thus, cancer cells with a relatively low content of hMSH2 would be unable to increase DNA damage to an extent sufficient to trigger cell death.

In conclusion, our study suggests a possible involvement of hMSH2 in ovarian cancer cell biology and in influencing the susceptibility to chemotherapeutic agents whereas the role of GTBP remains unclear. However, further studies are required to allow a better characterization of the function of the MMR system in human cancer.

### ACKNOWLEDGEMENTS

The authors are grateful to Dr Josef Jiricny for useful suggestions and reviewing the manuscript and to Dr Margherita Bignami for helpful discussions. Many thanks to Dr Marcello Anti and Dr Giovanni Cammarota for providing the specimens of normal human colonic mucosa used for the immunohistochemical analysis and to Dr Massimo Ercoli for the statistical analyses. This work was partially supported by Associazione Italiana Ricerca sul Cancro.

### REFERENCES

Acharya S, Wilson T, Gradia S, Kane MF, Guerrette S, Marsischky GT, Kolodner R and Fishel R (1996) hMSH2 forms specific mispairs-binding complexes with hMSH3 and hMSH6. *Proc Natl Acad Sci USA* **93**: 13629–13634

Aebi S, Kurdi-Haidar B, Gordon R, Cenni B, Zheng H, Fink D, Christen RD, Boland CR, Koi M, Fishel R and Howell SB (1996) Loss of DNA mismatch in acquired resistance to cisplatin. *Cancer Res* **56**: 3087–3090

Arzimanoglou II, Lallas T, Osborne M, Barber H and Gilbert F (1996) Microsatellite instability differences between familial and sporadic ovarian cancers. *Carcinogenesis* **17**: 1799–1804

Branch P, Hampson R and Karran P (1995) DNA mismatch binding defects, DNA damage tolerance, and mutator phenotypes in human colorectal carcinoma cell lines. *Cancer Res* **55**: 2304–2309

Boyer JC, Umar A, Risinger JI, Lipford JR, Kane M, Yin S, Barrett JC, Kolodner RD and Kunkel TA (1995) Microsatellite instability, mismatch repair deficiency, and genetic defects in human cancer cell lines. *Cancer Res* **55**: 6063–6070

Brown R, Hirst GL, Gallagher WM, McIlwarth AJ, Margison GP, van der Zee AGJ and Anthoney DA (1997) hMLH1 expression and cellular responses of ovarian tumour cells to treatment with cytotoxic anticancer agents. *Oncogene* **15**: 45–52

Dosch J, Christmann M and Kaina B (1998) Mismatch G-T binding activity and MSH2 expression is quantitatively related to sensitivity of cells to methylating agents. *Carcinogenesis* **19**: 567–573

Duckett DR, Drummond JT, Murchie AIH, Reardon JT, Sancar A, Lilley DMJ and Modrich P (1996) Human MutSα recognizes damaged DNA base pairs containing $O^6$-methylguanine, $O^4$-methylthymine, or the cisplatin-d (GpG) adduct. *Biochemistry* **93**: 6443–6447

Eshleman JR and Markowitz SD (1995) Microsatellite instability in inherited and sporadic neoplasms. *Curr Opin Oncol* **7**: 83–89

Fink D, Nebel S, Aebi S, Zheng H, Cenni B, Nehmé A, Christen RD and Howell SB (1996) The role of DNA mismatch repair in platinum drug resistance. *Cancer Res* **56**: 4881–4886

Glaab W and Tindall KR (1997) Mutation rate at the hprt locus in human cancer cell lines with specific mismatch repair-gene defects. *Carcinogenesis* **18**: 1–8

Hawn MT, Umar A, Carethers JM, Marra G, Kunkel TA, Boland CR and Koi M (1995) Evidence for a connection between the mismatch repair system and the G2 cell cycle checkpoint. *Cancer Res* **55**: 3721–3725

King BL, Carcangiu ML, Carter D, Kiechle M, Pfisterer J, Pfleiderer A and Kacinski BM (1995) Microsatellite instability in ovarian neoplasms. *Br J Cancer* **72**: 376–382

Kutteh WH and Kutteh CC (1992) Quantitation of tumor necrosis factor-alpha, interleukin-1 beta, and interleukin-6 in the effusions of ovarian epithelial neoplasms. *Am J Obstet Gynecol* **167**: 1864–1869

Leach FS, Polyak K, Burrell M, Johnson KA, Hill D, Dunlop G M, Wyllie A H, Peltomaki P, de la Chapelle A, Hamilton SR, Kinzler KW and Vogelstein B (1996) Expression of the human mismatch repair gene *hMSH2* in normal and neoplastic tissues. *Cancer Res* **56**: 235–240

Marra G, Chang CL, Laghi LA, Chanhan DP, Young D and Boland CR (1996) Expression of human MutS homolog 2 (hMSH2) protein in resting and proliferating cells. *Oncogene* **13**: 2189–2196

Mello JA, Acharya S, Fishel R and Essigmann JM (1996) The mismatch-repair protein hMSH2 binds selectively to DNA adducts of the anticancer drug cisplatin. *Chem Biol* **3**: 579–589

Modrich P and Lahue R (1996) Mismatch repair in replication fidelity, genetic recombination, and cancer biology. *Annu Rev Biochem* **65**: 101–133

Palombo F, Gallinari P, Iaccarino I, Lettieri T, Hughes M, D’Arrigo A, Truong O, Hsuan JJ and Jiricny (1995) GTBP, a 160-kilodalton protein essential for mismatch-binding activity in human cells. *Science* **268**: 1912–1914

Papadopoulos N, Nicolaides NC, Liu B, Parson R, Lengauer C, Palombo F, D’Arrigo A, Markowitz S, Willson JKV, Kinzler KW, Jiricny J and Vogelstein B (1995) Mutation of GTBP in genetically unstable cells. *Science* **268**: 1915–1917

Scambia G, Ranelletti FO, Benedetti Panici P, Piantelli M, De Vincenzo R, Bonanno G, Ferrandina G, Isola G and Mancuso S (1992) Synergistic antiproliferative activity of tamoxifen and cisplatin on primary ovarian tumours. *Eur J Cancer* **28A**: 1885–1889

 *© 1999 Cancer Research Campaign*

Serov SF and Scully R (1973) Histological typing of ovarian tumours. In: *International Histological Classification of Tumours*. World Health Organization: Geneva

Umar A, Boyer JC, Thomas DC, Nguyen DC, Risinger JI, Boyd J, Yonov Y, Perucho M and Kunkel TA (1994) Defective mismatch repair in extracts of colorectal and endometrial cancer cell lines exhibiting microsatellite instability. *J Biol Chem* **269**: 14367–14370

Wilson TM, Ewel A, Duguid JR, Ebel JN, Lescoe MK, Fishel R and Kelley MR (1995) Differential cellular expression of the human MSH2 repair enzyme in small and large intestine. *Cancer Res* **55**: 5146–5150

World Health Organization (1979) *WHO Handbook for Reporting Results of Cancer Treatment*. WHO: Geneva

Yamada M, O'Regan E, Brown R and Karran P (1997) Selective recognition of a cisplatin-DNA adduct by human mismatch repair proteins. *Nucleic Acids Res* **25**: 491–495

*© 1999 Cancer Research Campaign*
